# GENE-OBESOGENIC ENVIRONMENT INTERACTIONS ON BODY MASS INDICES ACROSS RACE AND SEX AMONG OLDER ADULTS

**DOI:** 10.1093/geroni/igz038.3155

**Published:** 2019-11-08

**Authors:** Mika D Thompson, Catherine M Pirkle, Fadi Youkhana, Yanyan Wu

**Affiliations:** University of Hawai‘i at Mānoa, Honolulu, Hawaii, United States

## Abstract

Gene-obesogenic environment interactions influence body mass index (BMI) across the life-course; however, limited research examines how these interactions may differ by race and sex. Utilizing mixed-effects models, we examined interaction effects of polygenic risk score (PGS) generated from 97 single nucleotide polymorphisms, and environmental factors, including age and physical activity, on measured longitudinal BMI from the Health and Retirement Study (HRS). HRS is a population representative survey study of older adults aged 50-years or older in the U.S. This study used a sub-sample of genotyped Black (N=1,796) and White (N=4,925) males and females. The association between PGS and mean BMI weakened as individuals aged among White males (Pinteraction=0.038) and White females (Pinteraction=0.054). The mean BMI difference between the highest and lowest PGS quintiles was 4.25 kg/m2 among 50-year old White males but 3.11 kg/m2 among the 70-year old’s, i.e. a decrease of 1.14 kg/m2 (95%CI: -0.06,2.65) over 20 years. Similarly, the decrease among 50- and 70-year old White females was 1.33 kg/m2 (95%CI: 0.07,3.45). Additionally, the association between physical activity and BMI was stronger among White females with higher PGS (Pinteraction=0.038). Vigorous physical activity (compared to never) was associated with a 1.71 kg/m2 (95%CI: 1.08,2.35) decrease in mean BMI among those in the highest PGS quintile, compared to a 0.80 kg/m2 (95%CI: 0.32,1.27) decrease among those in the lowest. Overall, we found unique interaction effects across race and sex within an older population; findings reinforce the importance of physical activity among those with an elevated genetic risk.

